# Normalization of elevated preoperative serum creatinine and acute kidney injury after cardiac surgery: a retrospective cohort study

**DOI:** 10.1038/s41598-025-13719-4

**Published:** 2025-07-31

**Authors:** Bo Jiang, Genshen Zhen, Haiping Yang, Yi Hao, Meiping Wang, Zhenhua Zhang, Lin Chen, Ning He, Yueling Chen, Li Jiang

**Affiliations:** 1 Intensive Critical Unit, Beijing Luhe Hospital, Capital Medical University, Beijing, China; 2 Department of Cardiac Surgery, Beijing Luhe Hospital, Capital Medical University, Beijing, China; 3 Intensive Critical Unit, Xuanwu Hospital, Capital Medical University, Beijing, China

**Keywords:** Acute kidney injury, Cardiac surgery, Preoperative assessment, Acute kidney injury, Risk factors

## Abstract

Preoperative kidney dysfunction is a predictor of acute kidney injury (AKI) following cardiac surgery, limited information exists regarding the impact of preoperative serum creatinine (sCr) change on AKI. This study aims to examine the association between the normalization of elevated preoperative sCr and postoperative AKI, as well as its severity and duration. This retrospective cohort study included patients undergoing open-heart surgery. Patients were categorized into three groups based on preoperative sCr change (ΔScr): the Stable sCr group (maximum ΔScr < 0.3 mg/dL throughout the preoperative period), the Normalized sCr group (maximum ΔScr ≥ 0.3 mg/dL followed by normalization to < 0.3 mg/dL within 48 h pre-surgery), and the Worsened sCr group (maximum ΔScr ≥ 0.3 mg/dL, remaining ≥ 0.3 mg/dL within 48 h pre-surgery). Multivariable logistic regression was used to evaluate the association between preoperative sCr change and postoperative AKI, severe AKI, and persistent AKI. To control for selection bias, propensity score matching (1:3) was used by matching covariates between the Normalized sCr and the Stable sCr group. Of the 560 patients included, 40.2% developed AKI. In the Normalized sCr group, the rate of AKI was 61.2%, severe AKI 22.4%, and persistent AKI 34.7%. Multivariable logistic regression analyses revealed the Normalized group was associated with higher risk of postoperative AKI (adjusted OR, 2.51; 95% CI, 1.30‒4.85, *p* = 0.006), severe AKI (adjusted OR, 3.40; 95% CI, 1.37–8.45, *p* = 0.008) and persistent AKI (adjusted OR, 2.87; 95% CI 1.36‒6.05, *p* = 0.006). After propensity matching, 184 patients were matched (46 in the Normalized sCr group and 138 in the Stable sCr group). The Normalized sCr group were still associated with risk of AKI (adjusted OR, 2.77; 95% CI, 1.34–5.73, *p* = 0.006), severe AKI (adjusted OR, 4.10; 95% CI, 1.32–12.71, *p* = 0.015) and persistent AKI (adjusted OR, 2.72; 95% CI, 1.21–6.15, *p* = 0.016). Normalization of preoperative sCr following an initial elevation was associated with higher risks of AKI, as well as AKI severity and duration.

**Supplementary Information:**

The online version contains supplementary material available at 10.1038/s41598-025-13719-4.

## Introduction

Acute kidney injury (AKI) is a common and serious complication of cardiac surgery that has a significant impact on patient morbidity and mortality^1–3^. Even patients with full kidney function recovery experience a heightened risk of CKD and mortality in the following years^4–6^. Limited availability of effective preventive interventions remains a significant challenge in mitigating the development of AKI. Given the high morbidity and mortality associated with AKI and the lack of targeted therapies, early detection is crucial.

Preoperative kidney dysfunction is a prevalent concern in patients undergoing cardiac surgery^7^. Elevated preoperative serum creatinine (sCr) is associated with an increased risk of developing AKI following cardiac surgery^8–10^. However, the effects of changes in preoperative sCr levels on postoperative AKI remain poorly understood. In particular, whether the normalization of elevated preoperative sCr levels impacts postoperative AKI has not been thoroughly investigated.

In this study, we aimed to examine the association between the normalization of elevated preoperative sCr levels and AKI, its severity, and duration in patients undergoing open-heart surgery.

## Methods

### Study population

This retrospective observational cohort study was carried out at Beijing Luhe Hospital, Capital Medical University, from January 2015 to December 2021. The inclusion criteria for eligible patients were: (1) aged 18 years or older, (2) underwent open-heart surgery, and (3) admission to the intensive care unit (ICU) post-surgery. Patients were excluded if they: (1) had emergency surgery, (2) had a baseline estimated glomerular filtration rate (eGFR) below 60 mL/min/1.73 m² [12], (3) had only one preoperative sCr measurement without available preadmission creatinine values, or (4) died or were discharged less than 24 h after surgery. The study protocol and the waiver of informed consent were approved by the Medical Ethics Committee of Beijing Luhe Hospital, Capital Medical University (approval reference number: 2022–KY–070).

### Data collection

Data on patient demographics, comorbidities, and preoperative medications were collected. Additionally, details on surgical procedures, length of ICU and hospital stays, and in-hospital mortality were recorded. The European System for Cardiac Operative Risk Evaluation II (EuroSCORE II), a predictive model for operative mortality in cardiac surgery, was calculated^11^. Cardiac function was assessed according to the New York Heart Association (NYHA) classification^12^. The sCr levels were tracked from admission through to discharge.

### Preoperative sCr change

The preoperative sCr change (ΔScr) was defined as the difference between preoperative sCr values and baseline sCr. Patients were categorized into three groups based on the ΔScr at its maximum and within 48 h before surgery: Stable sCr Group, with a maximum ΔScr of < 0.3 mg/dL throughout the preoperative period; Normalized sCr Group, with a maximum ΔScr of ≥ 0.3 mg/dL followed by normalization to < 0.3 mg/dL within 48 h before surgery; and Worsened sCr Group, with a maximum ΔScr of ≥ 0.3 mg/dL and remaining ≥ 0.3 mg/dL within 48 h before surgery. The 0.3 mg/dL threshold is based on the KDIGO definition of AKI, which identifies this level of increase in sCr as an indicator of AKI^13^. Baseline sCr was defined as the lowest sCr level recorded within the 6 months preceding hospital admission or, if unavailable, the sCr level at admission^14^. The maximum preoperative sCr level was recorded within 30 days before the procedure.

### Outcomes

The primary outcome was AKI within seven days following surgery. Secondary outcomes included severe AKI and persistent AKI postoperatively. AKI was defined as an increase in sCr ≥ 0.3 mg/dL or ≥ 1.5× baseline within 48 h or a urine output < 0.5 mL/kg/h for 6 h or more. KDIGO stage 1 was defined as an increase in sCr of 1.5 to 1.9× baseline or an absolute increase of ≥ 0.3 mg/dL within 48 h or a urine output < 0.5 mL/kg/h for 6 to 12 h. KDIGO stage 2 was defined as an increase in sCr of 2.0 to 2.9× baseline or a urine output < 0.5 mL/kg/h for ≥ 12 h, and KDIGO stage 3 was defined as an increase sCr of 3.0× baseline or an absolute increase of ≥ 4.0 mg/dL or initiation of CKRT or urine output < 0.3·mL/kg/h for ≥ 24 h or anuria for ≥ 12 h^13^. Severe AKI was defined as KDIGO stages 2 or 3. Persistent AKI is characterized by the continuance of AKI by sCr criteria beyond 48 h from AKI onset^15^.

### Statistical analysis

Continuous data were reported as medians with 25th and 75th percentiles. while categorical data were reported as counts and percentages. The nonparametric Wilcoxon rank-sum test was used to compare continuous variables, and the Kruskal-Wallis test was employed to compare more than two groups because none of the variables met the normality assumptions for parametric tests. Categorical variables were evaluated using the χ2 test and two-sided Fisher’s exact test as appropriate. To identify factors associated with the maximum ΔsCr, a generalized linear model was constructed, with the link function specified as family = Gamma(log). The model included demographics, comorbidities, and preoperative medications as explanatory variables. Multivariable logistic regression analysis was performed to evaluate the association between preoperative sCr changes and outcomes, including AKI, severe AKI, and persistent AKI. Adjusted variables included sex, age, baseline eGFR, hypertension, diabetes mellitus, NYHA classification, use of contrast agents, ACEIs or ARBs, diuretics, statins, NSAIDs, cardiopulmonary bypass, type of surgery, surgery duration, and intraoperative blood transfusion volume. To address selection bias, conditional logistic regression was performed on a propensity score-matched cohort that included patients in the Normalized sCr group and the Stable sCr group. To maintain coherence with the study aim, the worsened sCr group was not included in the matching process. Propensity score matching was performed with a 1:3 match, using the nearest neighbor method with a caliper size of 0.2 standard deviations, without replacement. The variables included in the propensity score matching were age, gender, baseline eGFR, EuroSCOREII, hypertension, diabetes mellitus, NYHA class, use of contrast agents, ACEIs or ARBs, diuretics, statins, NSAIDs, cardiopulmonary bypass, type of surgery, surgery duration, and intraoperative blood transfusion volume. Covariate balance was assessed using standardized mean differences (SMD), with post-matching SMD < 0.1 indicating good covariate balance between groups. Statistical analyses were performed using R software (ver. 4.1.2; R Foundation for Statistical Computing, Vienna, Austria). A two-tailed P value of less than 0.05 was considered statistically significant for all analyses.

## Results

### Study population

**Fig. 1:**
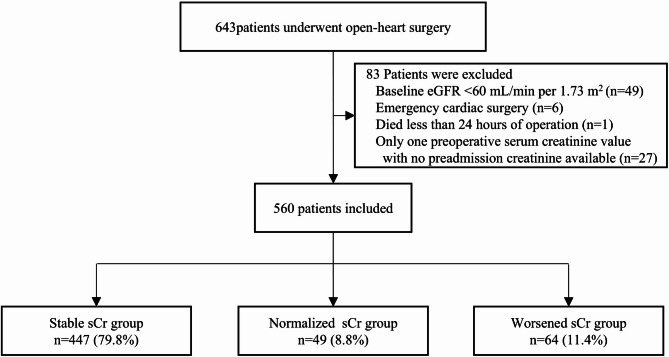
Flowchart of inclusion and exclusion. *eGFR* estimated glomerular filtration rate.

During the study period, 560 patients were included in the final analysis (Fig. 1). Baseline characteristics are presented in Table 1. 63.4% were male, with a median age of 64 [57–68] years. 30.5% of the patients were classified as NYHA class III/IV, with a median preoperative length of hospital stay of 14 [9–21] days. The number of preoperative serum creatinine measurements was 4 [3–5]. The Normalized sCr group and Worsened sCr group had a higher prevalence of NYHA class III/IV, and longer preoperative hospital stays compared to the Stable sCr group. They also experienced longer surgery times, and extended postoperative hospital and ICU stays.

**Table 1:** Characteristics of Patients.

Characteristic	All subjects (*n* = 560)	Stable sCr (*n* = 447)	Normalized sCr (*n* = 49)	*P* value*	Worsened sCr (*n* = 64)	*P* value**
Age, year	64 [57–68]	64 [57–68]	64 [56–69]	0.905	64 [60–69]	0.200
Male	355 (63.4)	282 (63.1)	33 (67.3)	0.557	40 (62.5)	0.008
EuroSCOREII	2.0 [1.0–3.0]	2.0 [1.0–3.0]	3.0 [1.0–4.0]	0.120	3.0 [2.0–4.0]	0.013
Hypertension	333 (59.5)	257 (57.5)	31 (63.3)	0.437	45 (70.3)	0.051
Diabetes mellitus	163 (29.1)	130 (29.1)	13 (26.5)	0.708	20 (31.2)	0.127
NYHA class III/IV	171 (30.5)	117 (26.2)	26 (53.1)	< 0.001	28 (43.8)	0.004
ACEIs or ARBs	265 (47.3)	220 (49.2)	18 (36.7)	0.097	27 (42.2)	0.293
Diuretics	331 (59.1)	267 (59.7)	28 (57.1)	0.726	36 (56.2)	0.596
Statins	282 (50.4)	227 (50.8)	22 (44.9)	0.434	33 (51.6)	0.907
NSAIDs	274 (48.9)	219 (49)	23 (46.9)	0.785	32 (50)	0.880
Contrast agent ^a^	474 (84.6)	376 (84.1)	42 (85.7)	0.771	56 (87.5)	0.484
Baseline eGFR, mL/min /1.73 m^2^	89.8 [80.0–97.7]	91.1 [82.5–98.2]	86.7 [78.5–97.3]	0.150	80.6 [71.5–93.1]	< 0.001
Maximum ΔScr, mg/dL^b^	0.15 [0.06–0.26]	0.12 [0.03–0.18]	0.40 [0.33–0.55]	< 0.001	0.51 [0.41‒0.76]	< 0.001
Maximum ΔScr prior to surgery, day	6 [3–12]	6 [3–11]	11 [7–17]	< 0.001	4 [2–12]	0.246
Preoperative length of stay, day	14 [9–21]	13 [8–20]	20 [15–27]	< 0.001	18 [13–28]	< 0.001
Type of surgery				0.075		0.001
Coronary artery bypass graft surgery	279 (49.9)	238 (53.2)	20 (40.8)		21 (32.8)	
Valve surgery	218 (38.9)	157 (35.2)	25 (51.0)		36 (56.3)	
Combined	41 (7.3)	30 (6.7)	4 (8.2)		7 (10.9)	
Other	22 (3.9)	22 (4.9)	0 (0.0)		0 (0.0)	
Surgery time, min	314 [260–365]	305 [257–360]	325 [299–385]	0.016	337 [284–394]	0.003
Cardiopulmonary bypass	300 (53.6)	223 (49.9)	31 (63.3)	0.075	46 (71.9)	< 0.001
Intraoperative blood product transfusion volume, mL	1105 [252–1686]	1010 [206–1647]	1320 [500–2000]	0.042	1408 [1053–1818]	0.001
IABP/ECMO	59 (10.5)	41 (9.2)	9 (18.4)	0.074	9 (14.1)	0.218
ICU length of stay, day	2.0 [2.0–4.0]	2.0 [2.0–4.0]	3.0 [2.0–6.0]	0.007	3.0 [2.0–5.0]	< 0.001
Hospital length of stay, day	27 [20–35]	25 [19–33]	33 [28–44]	< 0.001	33 [24–40]	< 0.001

Data are reported as medians [25th-75th percentile] or numbers (percentage), where appropriate.

*EuroSCOREII* European System for Cardiac Operative Risk Evaluation, *ACEI* angiotensin-converting enzyme inhibitor, *ARB* angiotensin receptor blocker, *NYHA* New York Heart Association, *ICU* intensive care unit, *eGFR* estimated glomerular filtration rate, *ECMO* extracorporeal membrane oxygenation, *IABP* intra-aortic balloon pumping, *NSAID* non-steroidal anti-inflammatory drugs, *IQR* interquartile range, *sCr* serum creatinine.

^a^Contrast agent was recorded within one month before surgery.

^b^Maximum ΔScr defined as the difference between maximum preoperative serum creatinine values and baseline serum creatinine.

*P value versus Stable sCr group.

**P value versus Stable sCr group.

### Change in preoperative serum creatinine

The maximum ΔScr was defined as the difference between the maximum preoperative sCr value and the baseline sCr. The median maximum ΔScr was 0.15 [0.06–0.26] mg/dL. The Normalized sCr group and the Worsened sCr group exhibited higher maximum ΔScr compared to the Stable sCr group. Using a generalized linear model, NYHA class III/IV was associated with an increase in maximum ΔsCr (β = 0.147, *P* < 0.001).

The maximum ΔScr occurred at a median of 6 days [3–12] prior to surgery, as depicted in Supplementary Fig. 1. The Normalized sCr group demonstrated an earlier occurrence of maximum ΔScr compared to the Stable sCr group (11 days [7–17] vs. 6 days [3–11]; *P* < 0.001). No significant difference was observed for the Worsened sCr group. Stratification by the median timing of maximum ΔsCr showed that occurrences ≥ 7 days before surgery were linked to a higher prevalence of NYHA class III/IV, as detailed in Supplementary Table 1.

Postoperative AKI.

Among 560 patients, 225 (40.2%) developed AKI within 7 days post-surgery, including 57 (10.2%) with severe AKI, 107 (19.1%) with persistent AKI, and 18 (3.2%) requiring CRRT. The Normalized sCr and Worsened sCr groups showed higher incidences of AKI, severe AKI, and persistent AKI compared to the Stable sCr group (Supplementary Table 2).

The timing of maximum ΔsCr was linked to the development of AKI. Patients exhibiting an earlier maximum ΔsCr (≥ 7 days prior to surgery) showed a higher incidence of AKI compared to those with maximum ΔsCr within 7 days (*p* = 0.007). However, this association was not associated with severe AKI or persistent AKI (Supplementary Table 3).

### Association of preoperative sCr change with AKI

Multivariable logistic regression analyses demonstrated that the Normalized sCr group was independently associated with significantly elevated risks of AKI (adjusted OR: 2.51; 95% CI: 1.30–4.85, *p* = 0.006), severe AKI (adjusted OR: 3.40; 95% CI: 1.37–8.45, *p* = 0.008), and persistent AKI (adjusted OR: 2.87; 95% CI: 1.36–6.05, *p* = 0.006) compared to the Stable sCr group (Table 2). Similarly, the Worsened sCr groups exhibited notably higher risks of AKI (adjusted OR: 7.79; 95% CI: 3.77–16.11, *p* < 0.001), severe AKI (adjusted OR: 5.38; 95% CI: 2.46–11.78, *p* < 0.001), and persistent AKI (adjusted OR: 7.90; 95% CI: 4.10–15.25, < 0.001).

**Table 2:** Logistic regression analyses on AKI for changes in preoperative serum creatinine.

	AKI		Severe AKI		Persistent AKI	
	OR (95% CI)	P value	OR (95% CI)	P value	OR (95% CI)	P value
Unadjusted						
Stable sCr Group	1.00 (Reference)		1.00 (Reference)		1.00 (Reference)	
Normalized sCr Group	3.39 (1.85–6.23)	< 0.001	4.50 (2.07–9.78)	< 0.001	3.79 (1.97–7.27)	< 0.001
Worsened sCr Group	10.35 (5.25–20.41)	< 0.001	6.57 (3.39–12.74)	< 0.001	8.60 (4.88–15.17)	< 0.001
Adjusted*						
Stable sCr Group	1.00 (Reference)		1.00 (Reference)		1.00 (Reference)	
Normalized sCr Group	2.51 (1.30–4.85)	0.006	3.40 (1.37–8.45)	0.008	2.87 (1.36–6.05)	0.006
Worsened sCr Group	7.79 (3.77–16.11)	< 0.001	5.38 (2.46–11.78)	< 0.001	7.90 (4.10‒15.25)	< 0.001

*AKI* acute kidney injury, *sCr* serum creatinine.

*Multivariable logistic regression was adjusted for age, gender, hypertension, diabetes mellitus, NYHA class III/IV, ACEIs or ARBs, diuretics, statins, NSAIDs, contrast agent, baseline eGFR, type of surgery, surgery time, cardiopulmonary bypass, and intraoperative blood transfusion volume.

### Association normalized sCr group and AKI in propensity-matched cohort

To investigate selection bias and evaluate the association between the Normalized sCr and the risk of AKI, severe AKI, and persistent AKI after surgery, we conducted a cohort utilizing propensity score matching. Detailed characteristics after matching are depicted in Supplemental Table 4. The Normalized sCr group exhibited a significantly higher risk of AKI (adjusted OR, 2.77; 95% CI, 1.34‒5.73, *p* = 0.006) (Supplemental Table 5), severe AKI (adjusted OR, 4.10; 95% CI, 1.32–12.71, *p* = 0.015) (Supplemental Table 6), and persistent AKI (adjusted OR, 2.72; 95% CI, 1.21‒6.15, *p* = 0.016) (Supplemental Table 7) compared to the Stable sCr group in propensity matched cohort. This analysis confirmed that the risks of AKI, severe AKI, and persistent AKI remain elevated after preoperative sCr levels normalize following an initial rise.

## Discussion

This study found a significant association between preoperative sCr change and risk of postoperative AKI in patients undergoing open-heart surgery. Elevated and then normalized preoperative sCr were associated with higher risks of postoperative AKI, as well as increased AKI severity and duration.

Preoperative kidney dysfunction is common in cardiac surgery patients, with up to 78% of coronary artery bypass grafting patients exhibiting some degree of kidney impairment^7^. Several studies have demonstrated that elevated preoperative sCr (> 1.2 mg/dL) and reduced eGFR are significantly associated with an increased risk of postoperative AKI^8,9^. Moreover, dynamic changes in sCr offer a more accurate assessment of AKI risk compared to single measurements^16,17^. Griffin et al. reported that elevated preoperative sCr from baseline at the time of cardiac surgery was associated with severe postoperative complications, including mortality, infection, stage 3 AKI, and prolonged ICU stay^18^. Our study extends these findings by emphasizing the importance of preoperative sCr change in assessing AKI risk and its impact on postoperative kidney outcomes.

Preoperative elevation of sCr is closely associated with cardiac dysfunction. In our study, 30.5% of patients exhibited NYHA class III/IV, and a significant correlation was observed between NYHA class III/IV and an increase in preoperative maximum ΔsCr. This relationship highlights the complex cardiac-kidney interplay, particularly in advanced heart failure, where reduced renal perfusion, systemic inflammation, and hemodynamic instability contribute to preoperative kidney dysfunction^19^.

The timing of preoperative maximum ΔScr offers critical insights into kidney function dynamics and AKI development. The Normalized sCr group experienced an earlier occurrence of maximum ΔScr, with a median timing of 11 days prior to surgery, compared to 6 days in the Stable sCr group. This pattern indicates that patients in the Normalized sCr group tend to experience transient kidney dysfunction earlier in the preoperative period, followed by recovery. In contrast, the Worsened sCr group had a shorter interval between the occurrence of maximum ΔsCr and surgery, suggesting that kidney dysfunction had not yet recovered. Notably, preoperative normalization of sCr may reduce AKI risk compared to persistent kidney dysfunction, highlighting the significance of preoperative sCr fluctuations in determining postoperative kidney outcomes.

However, the Normalized sCr group was at a higher risk of postoperative AKI, severe AKI, and persistent AKI compared to the Stable sCr group. This increased risk may be due to several factors. Firstly, normalization of preoperative sCr may reflect temporary hemodynamic improvement rather than full kidney recovery. Although this condition may resolve before surgery, underlying damage and stress on the kidneys may persist, making them more susceptible to AKI during and after surgery^20,21^. Secondly, sCr is not a sensitive marker for subtle kidney impairment, as significant increases typically occur only after more than 50% of kidney function is lost. The normalization of sCr may mask underlying kidney damage or diminished reserve, as kidneys function usually maintain through compensatory hyperfiltration in the remaining healthy nephrons during insults which consequently impair kidney functional reserve^22^. Husain-Syed et al. showed that a persistent decrease in kidney functional reserve occurs in patients with AKI even after clinical recovery^23^. This reduction in kidney functional reserve may indicate incomplete kidney recovery, predisposing individuals to latent AKI susceptibility^24^. Even mild surgical stress can further increase the risk of AKI when combined with these factors^25^.

Our study underscores the clinical significance of preoperative sCr fluctuations. While normalization of sCr is generally considered a marker of kidney recovery, our findings indicate that it may still be linked to an elevated risk of postoperative AKI compared to stable sCr, suggesting that kidney recovery could be incomplete^26^. Consequently, a thorough evaluation of normalized sCr during preoperative assessment, along with analysis of underlying kidney vulnerabilities, is essential. In addition, the AKI incidence in our cohort was 40.2%. While this falls within the reported range of 5–43%^27,28^, it remains relatively high. One contributing factor may be the high proportion (30.5%) of patients with NYHA class III/IV, a population known to have increased susceptibility to AKI due to impaired hemodynamics, venous congestion, and reduced renal perfusion. Notably, we also found a significant association between preoperative cardiac dysfunction and sCr fluctuation, further emphasizing the importance of evaluating sCr dynamics in this high-risk patient. To enhance risk stratification, incorporating biomarkers such as KIM-1 and NGAL^29,30^which can detect subtle kidney injury, may provide additional insight into kidney vulnerability. Furthermore, longitudinal studies are crucial for examining AKI progression across different time points following sCr normalization. A deeper understanding of these patterns will be crucial for optimizing surgical timing, refining perioperative management strategies, and ultimately improving patient outcomes.

Several limitations should be acknowledged. First, the retrospective design constrains the ability to infer causality and increases the risk of bias. Since exposures and outcomes were neither assigned nor prospectively measured, the observed associations may have been influenced by unmeasured confounding. Although adjustments were made for several known variables, other factors such as intraoperative hemodynamics, fluid balance, and nephrotoxic drug use were not captured and may have contributed to residual bias. Moreover, the lack of daily preoperative sCr, especially outside of the ICU, further compromised the accuracy of exposure classification. Second, the selection of baseline sCr may introduce bias. Currently, there is no universally established definition for baseline sCr. Existing studies have adopted a variety of reference time points, ranging from the day of surgery to measurements taken 3 to 12 months prior to admission. Ideally, baseline sCr should represent a patient’s stable renal function, preferably derived from serial outpatient assessments. However, such data are frequently unavailable in real-world clinical settings, making it difficult to determine an accurate baseline and potentially biasing the results^31^. Third, the exclusion of patients with only a single preoperative sCr measurement and those with preexisting chronic kidney disease (CKD) may have introduced selection bias and reduced the generalizability of our findings, as these criteria likely favored higher-risk individuals and excluded a substantial portion of the population at risk for AKI. Fourth, sCr is an indirect and relatively insensitive marker for detecting subclinical kidney injury, as its concentration can be affected by various non-renal factors, including volume status, muscle mass, and metabolic state. Furthermore, the lack of validated biomarkers specific to early kidney injury, such as neutrophil gelatinase-associated lipocalin and kidney injury molecule 1, further limits the ability to mechanistically interpret the observed sCr changes. Finally, the clinical utility of monitoring sCr trajectories to guide surgical timing remains uncertain. Prospective studies incorporating serial sCr measurements and biomarker assessment are needed to clarify the underlying pathophysiology and determine their relevance for perioperative decision-making.

## Conclusions

This study highlights the relationship between preoperative sCr change and the development of AKI. Normalization of preoperative sCr following an initial elevation remains associated with higher risks of AKI, as well as AKI severity and duration.

## Supplementary Information

Below is the link to the electronic supplementary material.

Supplementary Material 1

## Data Availability

The datasets generated during and/or analysed during the current study are available from the corresponding author on reasonable request.
