# Supplementary material for: Normalization of elevated preoperative serum creatinine and acute kidney injury after cardiac surgery: a retrospective cohort study
Source: Sci Rep. 2025 Jul 31;15:27933. doi: 10.1038/s41598-025-13719-4 (PMC12313847; doi:10.1038/s41598-025-13719-4)
Supplement: Supplementary file 1 — Supplementary Material 1 [file 41598_2025_13719_MOESM1_ESM.docx]

**Supplementary material for: Normalization of Elevated Preoperative Serum Creatinine and Acute Kidney Injury after Cardiac Surgery: A Retrospective Cohort Study**

Supplementary Figure 1. Distribution of maximum ΔScr.

Supplementary Table 1 Characteristics of patients grouped by timing of maximum Scr

Supplementary Table 2. Incidence of AKI grouped by preoperative creatinine patterns

Supplementary Table 3. Incidence of AKI grouped by creatinine patterns and timing of maximum Scr

Supplementary Table 4. Perioperative demographic and risk factors in propensity-matched cohort

Supplementary Table 5. Association between Normalized sCr group and AKI in propensity–matched cohort

Supplementary Table 6. Association between Normalized sCr group and severe AKI in propensity-matched cohort

Supplementary Table 7. Association between Normalized sCr group and persistent AKI in propensity-matched cohort


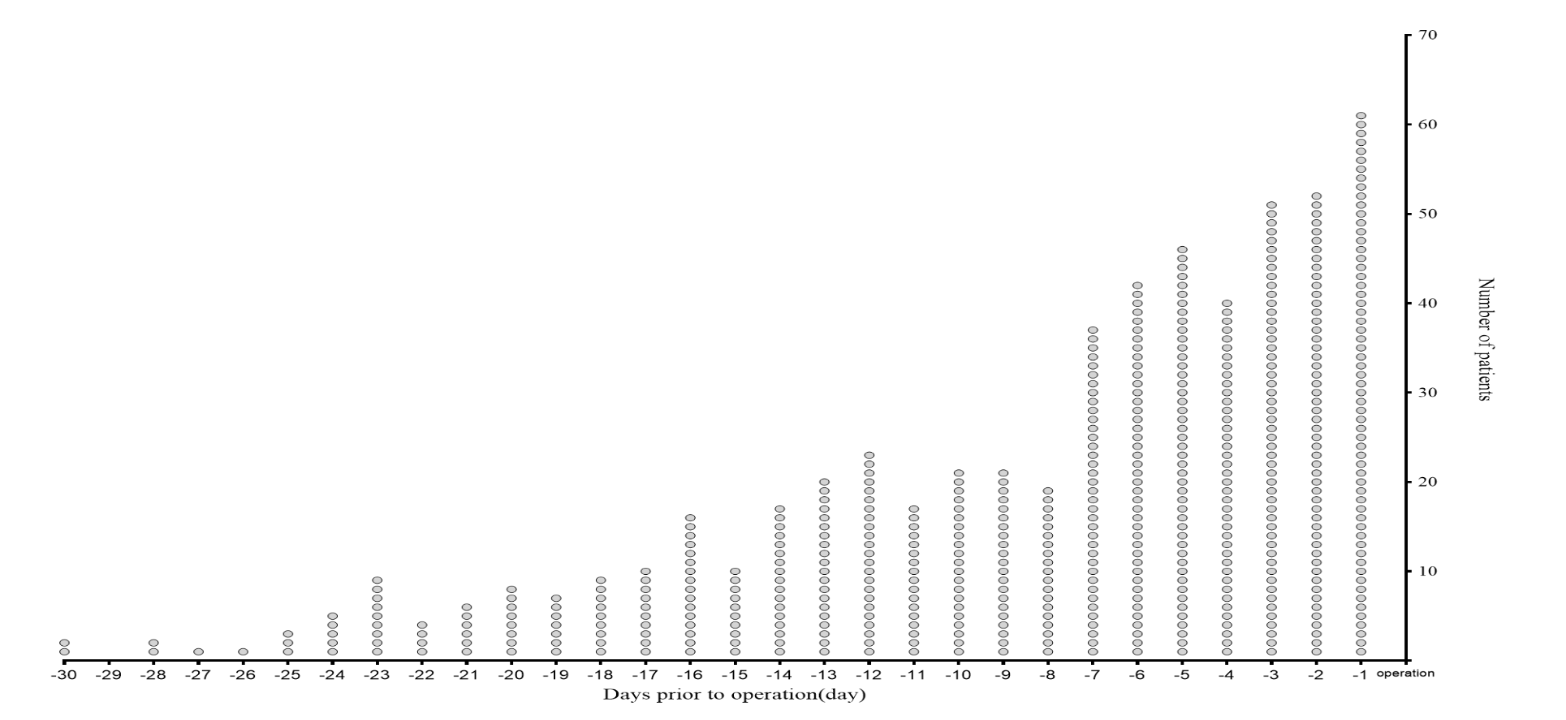


Supplementary Figure 1. Distribution of maximum ΔScr. Maximum ΔScr was defined as the difference between the maximum preoperative sCr values and baseline sCr.

Supplementary Table 1. Characteristics of patients grouped by timing of maximum ΔScr

| Characteristic | All subjects  (n=560) | Timing of maximum ΔScr | | P value |
| --- | --- | --- | --- | --- |
|  |  | < 7days (n=300) | ≥7 days (n=260) |  |
| Age, year | 64 [57–68] | 64 [57–68] | 64 [57–68] | 0.757 |
| Male | 355 (63.4) | 193 (64.3) | 162 (62.3) | 0.62 |
| EuroSCOREII | 2.0 [1.0–3.0] | 2.0 [1.0–3.0] | 2.0 [1.0–4.0] | 0.14 |
| Hypertension | 333 (59.5) | 169 (56.3) | 164 (63.1) | 0.437 |
| Diabetes mellitus | 163 (29.1) | 78 (26) | 85 (32.7) | 0.082 |
| NYHA class III/IV | 171 (30.5) | 73 (24.3) | 98 (37.7) | < 0.001 |
| ACEIs or ARBs | 265 (47.3) | 147 (49) | 118 (45.4) | 0.393 |
| Diuretics | 331 (59.1) | 179 (59.7) | 152 (58.5) | 0.772 |
| Statins | 282 (50.4) | 150 (50) | 132 (50.8) | 0.856 |
| NSAIDs | 274 (48.9) | 146 (48.7) | 128 (49.2) | 0.894 |
| Contrast agent ^a^ | 474 (84.6) | 250 (83.3) | 224 (86.2) | 0.356 |
| Baseline eGFR, mL/min /1.73 m^2^ | 89.8 [80.0–97.7] | 91.0 [80.8–98.9] | 89.3 [79.3–97.1] | 0.216 |
| Maximum ΔScr, mg/dL^b^ | 0.15 [0.06–0.26] | 0.15 [0.06–0.27] | 0.15 [0.08–0.26] | 0.762 |
| Preoperative length of stay, day | 14 [9–21] | 11 [7–17] | 18 [13–27] | < 0.001 |
| Type of surgery |  |  |  | 0.890 |
| Coronary artery bypass graft surgery | 279 (49.9) | 152 (50.7) | 127 (48.8) |  |
| Valve surgery | 218 (38.9) | 113 (37.7) | 105 (40.4) |  |
| Combined | 41 (7.3) | 22 (7.3) | 19 (7.3) |  |
| Other | 22 (3.9) | 13 (4.3 | 9 (3.9) |  |
| Surgery time, min | 314 [260–365] | 308 [259–359] | 320 [270–375] | 0.031 |
| Cardiopulmonary bypass | 300 (53.6) | 157 (52.3) | 143 (55.0) | 0.528 |
| Intraoperative blood product transfusion volume, mL | 1105 [252–1686] | 1100 [269–1679] | 1120 [230–1713] | 0.742 |
| IABP/ECMO | 59 (10.5) | 27 (9.0) | 32 (12.3) | 0.204 |
| ICU length of stay, day | 2 [2–4] | 2 [2–4] | 3 [2–4] | 0.023 |
| Hospital length of stay, day | 27 [20–35] | 23 [18–30] | 30 [23–41] | <0.001 |

Data are reported as medians [25th-75th percentile] or numbers (percentage), where appropriate.

Abbreviations: EuroSCOREII, European System for Cardiac Operative Risk Evaluation II; ACEI, angiotensin-converting enzyme inhibitor; ARB, angiotensin receptor blocker; NYHA, New York Heart Association; ICU, intensive care unit; eGFR, estimated glomerular filtration rate; ECMO, extracorporeal membrane oxygenation; IABP, intra-aortic balloon pumping; NSAID, non-steroidal anti-inflammatory drugs.

^a^ Contrast agent was recorded within one month before surgery.

^b^ Maximum ΔScr defined as the difference between maximum preoperative serum creatinine values and baseline serum creatinine

Supplementary Table 2 Incidence of AKI grouped by preoperative creatinine patterns

|  | Stable sCr Group | Normalized sCr Group | P-value | Worsened sCr Group | P-value |
| --- | --- | --- | --- | --- | --- |
| AKI, n (%) | 142 (31.8) | 30 (61.2) | <0.001 | 53 (82.8) | <0.001 |
| Severe AKI, n (%) | 27 (6.0) | 11 (22.4) | <0.001 | 19 (29.7) | <0.001 |
| Persistent AKI, n (%) | 55 (12.3) | 17 (34.7) | 0.001 | 35 (54.7) | <0.001 |

Abbreviations: AKI, acute kidney injury; sCr, serum creatinine.

Supplementary Table 3. Incidence of AKI grouped by creatinine patterns and timing of maximum ΔScr

|  | Timing of maximum ΔScr | | P value |
| --- | --- | --- | --- |
|  | < 7days | ≥7 days |  |
| AKI, % |  |  | 0.007 |
| Stable sCr group | 27.0 (66/244) | 37.4 (76/203) |  |
| Normalized sCr group | 70.0 (7/10) | 58.9 (23/39) |  |
| Worsened sCr group | 76.3 (29/38) | 92.3 (24/26) |  |
| Severe AKI, % |  |  | 0.477 |
| Stable sCr group | 5.6 (14/252) | 6.7 (13/195) |  |
| Normalized sCr group | 30.0 (3/10) | 20.5 (8/39) |  |
| Worsen sCr group | 28.9 (11/38) | 30.8 (8/26) |  |
| Persistent AKI, % |  |  | 0.251 |
| Stable sCr group | 10.7 (27/252) | 14.4 (28/195) |  |
| Normalized sCr group | 40.0 (4/10) | 33.3 (13/39) |  |
| Worsen sCr group | 55.3 (21/38) | 53.8 (14/26) |  |

Abbreviations: AKI, acute kidney injury; sCr, serum creatinine.

Supplementary Table 4. Perioperative demographic and risk factors in propensity-matched cohort

| Variable | Before match | | |  | After match | | |
| --- | --- | --- | --- | --- | --- | --- | --- |
|  | Stable sCr group（n=447） | Normalized sCr group (n=49) | SMD |  | Stable sCr group  (n=138) | Normalized sCr group  (n=46) | SMD |
| Age, year | 64 [57‒68] | 64 [56‒69] | 0.057 |  | 64 [57‒68] | 64 [56‒69] | 0.028 |
| Euroscore II | 2.0 [1.0‒3.0] | 3.0 [1.0‒4.0] | 0.260 |  | 2.0 [1.0‒4.0] | 2.0 [1.0‒3.8] | 0.022 |
| Male | 282 (63.1) | 33 (67.3) | 0.09 |  | 98 (71.0) | 31 (67.4) | 0.079 |
| Contrast agent ^a^ | 376 (84.1) | 42 (85.7) | 0.045 |  | 119 (86.2) | 41 (89.1) | 0.088 |
| Hypertension | 257 (57.5) | 31 (63.3) | 0.118 |  | 81 (58.7) | 29 (63.0) | 0.089 |
| Diabetes | 130 (29.1) | 13 (26.5) | 0.057 |  | 31 (22.5) | 12 (26.1) | 0.085 |
| NYHA class III/IV | 117 (26.2) | 26 (53.1) | 0.572 |  | 69 (50.0) | 23 (50.0) | <0.001 |
| Baseline eGFR, mL/min /1.73 m^2^ | 91.1 [82.5‒98.2] | 86.7 [78.5‒97.3] | 0.249 |  | 87.7 [78.4‒96.8] | 86.6 [78.4‒96.3] | 0.083 |
| Type of surgery |  |  | 0.446 |  |  |  | 0.027 |
| Coronary artery bypass graft surgery | 278 (49.6) | 20 (40.8) |  |  | 61 (44.2) | 20 (43.5) |  |
| Valve surgery | 212 (37.9) | 25 (51.0) |  |  | 66 (47.8) | 22 (47.8) |  |
| Combined | 48 (8.6) | 4 (8.2) |  |  | 11 (8.0) | 4 (8.7) |  |
| Surgery time, min | 305 [257‒360] | 325 [299‒385] | 0.356 |  | 326 [270‒382] | 325 [293‒374] | 0.031 |
| Cardiopulmonary bypass | 223 (49.9) | 31 (63.3) | 0.272 |  | 83 (60.1) | 28 (60.9) | 0.015 |
| Intraoperative blood product transfusion, mL | 1010 [206‒1647] | 1320 [500‒2000] | 0.267 |  | 1310 [446‒1760] | 1275 [406‒1983] | 0.043 |
| Stains | 227 (50.8) | 22 (44.9) | 0.118 |  | 67 (48.6) | 20 (43.5) | 0.102 |
| ACEI or ARB | 220 (49.2) | 18 (36.7) | 0.254 |  | 57 (41.3) | 18 (39.1) | 0.044 |
| NSIAD | 219 (49.0) | 23 (46.9) | 0.041 |  | 65 (47.1) | 21 (45.7) | 0.029 |
| Diuretics | 267 (59.7) | 28 (57.1) | 0.053 |  | 74 (53.6) | 27 (58.7) | 0.102 |

Data are reported as medians [25th-75th percentile] or numbers (percentage), where appropriate.

Abbreviations: EuroSCOREII, European System for Cardiac Operative Risk Evaluation II; ACEI, angiotensin-converting enzyme inhibitor; ARB, angiotensin receptor blocker; NYHA, New York Heart Association; ICU, intensive care unit; eGFR, estimated glomerular filtration rate; NSAID, non-steroidal anti-inflammatory drugs.

^a^ Contrast agent was recorded within one month before surgery.

^b^ Maximum ΔScr defined as the difference between maximum preoperative serum creatinine values and baseline serum creatinine

Supplementary Table 5. Association between normalized sCr group and AKI in propensity–matched cohort

| Variable | unadjusted OR (95%CI) | P-value | Adjusted OR (95%CI) | P-value |
| --- | --- | --- | --- | --- |
| Stable sCr group | Reference |  | Reference |  |
| Normalized sCr group | 2.42 (1.23‒4.79) | 0.011 | 2.77 (1.34–5.73) | 0.006 |
| Baseline eGFR, mL/min /1.73 m^2^ | 0.99 (0.97–1.01) | 0.416 |  |  |
| Age, year | 1.01 (0.98‒1.04) | 0.701 |  |  |
| Male | 1.60 (0.83‒3.07) | 0.161 |  |  |
| Contrast agent | 1.72 (0.73‒4.17) | 0.214 |  |  |
| Hypertension | 1.39 (0.77–2.56) | 0.270 |  |  |
| Diabetes | 1.32 (0.66‒2.70) | 0.433 |  |  |
| NYHA class III/IV | 2.06 (1.13‒3.73) | 0.018 | 2.23(1.02‒4.89) | 0.046 |
| Type of surgery |  |  |  |  |
| Coronary artery bypass graft surgery | Reference | Reference |  |  |
| Valve surgery | 2.00 (1.07‒3.73) | 0.029 |  |  |
| Combined | 1.75 (0.57–5.33) | 0.325 |  |  |
| Surgery time, per 30 min | 1.13 (1.04‒1.24) | 0.005 |  |  |
| Cardiopulmonary bypass | 2.36 (1.27‒4.40) | 0.007 |  |  |
| Intraoperative blood product transfusion, per 100mL | 1.06 (1.03–1.10) | 0.001 |  |  |
| Stains | 1.58 (0.88‒2.85) | 0.127 |  |  |
| ACEI or ARB | 1.23 (0.68–2.22) | 0.503 |  |  |
| NSIAD | 1.64 (0.91‒2.96) | 0.098 |  |  |
| Diuretics | 0.71 (0.39–1.28) | 0.253 |  |  |

Supplementary Table 6. Association between normalized sCr group and severe AKI in propensity-matched cohort

| Variable | Unadjusted OR (95%CI) | P-value | Adjusted OR (95%CI) | P-value |
| --- | --- | --- | --- | --- |
| Stable sCr group | Reference |  | Reference |  |
| Normalized sCr group | 3.02 (1.09‒8.36) | 0.034 | 4.10 (1.32–12.71) | 0.015 |
| Baseline eGFR, mL/min /1.73 m^2^ | 0.97 (0.93‒1.01) | 0.147 |  |  |
| Age, year | 1.01 (0.96–1.07) | 0.747 |  |  |
| Male | 1.72 (0.63–4.76) | 0.291 |  |  |
| Contrast agent | 2.27 (0.67‒7.69) | 0.188 |  |  |
| Hypertension | 1.04 (0.38–2.86) | 0.933 |  |  |
| Diabetes | 1.41 (0.47–4.27) | 0.538 |  |  |
| NYHA class III/IV | 3.62 (1.13–11.56) | 0.030 | 4.98 (1.103–22.50) | 0.037 |
| Type of surgery |  |  |  |  |
| Coronary artery bypass graft surgery | Reference | Reference |  |  |
| Valve surgery | 3.04 (0.94‒9.84) | 0.064 |  |  |
| Combined | 1.37 (0.14–13.23) | 0.783 |  |  |
| Surgery time, per 30 min | 1.20 (1.06‒1.35) | 0.003 |  |  |
| Cardiopulmonary bypass | 3.37 (0.93–12.16) | 0.064 |  |  |
| Intraoperative blood product transfusion, per 100mL | 1.07 (1.02‒1.12) | 0.005 |  |  |
| Stains | 1.28 (0.47–3.49) | 0.624 |  |  |
| ACEI or ARB | 1.72 (0.63‒4.69) | 0.288 |  |  |
| NSIAD | 1.31 (0.48–3.57) | 0.591 |  |  |
| Diuretics | 0.92 (0.34‒2.49) | 0.865 |  |  |

Supplementary Table 7. Association between normalized sCr group and persistent AKI in propensity-matched cohort

| Variable | Unadjusted OR  (95%CI) | P‒value | Adjusted OR  (95%CI) | P‒value |
| --- | --- | --- | --- | --- |
| Stable sCr group | Reference |  | Reference |  |
| Normalized sCr group | 2.44 (1.12‒5.32) | 0.025 | 2.72 (1.21‒6.15) | 0.016 |
| Baseline eGFR, mL/min /1.73 m^2^ | 1.01 (0.98–1.04) | 0.488 |  |  |
| Age, year | 1.02 (0.98‒1.06) | 0.378 |  |  |
| Male | 1.10 (0.49‒2.44) | 0.825 |  |  |
| Contrast agent | 1.92 (0.74–5.00) | 0.18 |  |  |
| Hypertension | 1.52 (0.72‒3.23) | 0.265 |  |  |
| Diabetes | 1.99 (0.89–4.43) | 0.094 |  |  |
| NYHA class III/IV | 1.91 (0.90‒4.07) | 0.094 |  |  |
| Type of surgery |  |  |  |  |
| Coronary artery bypass graft surgery | Reference |  |  |  |
| Valve surgery | 1.54 (0.71‒3.34) | 0.276 |  |  |
| Combined | 1.25 (0.25–6.25) | 0.790 |  |  |
| Surgery time, per 30 min | 1.13 (1.03–1.25) | 0.011 |  |  |
| Cardiopulmonary bypass | 1.83 (0.82‒4.08) | 0.139 |  |  |
| Intraoperative blood product transfusion, per 100mL | 1.05 (1.01–1.08) | 0.015 |  |  |
| Stains | 1.07 (0.51–2.23) | 0.865 |  |  |
| ACEI or ARB | 1.48 (0.71‒3.11) | 0.298 |  |  |
| NSIAD | 1.26 (0.60–2.64) | 0.537 |  |  |
| Diuretics | 0.63 (0.30‒1.33) | 0.227 |  |  |
